# Bidirectional Microglia-Neuron Communication in the Healthy Brain

**DOI:** 10.1155/2013/456857

**Published:** 2013-09-02

**Authors:** Ukpong B. Eyo, Long-Jun Wu

**Affiliations:** Department of Cell Biology and Neuroscience, Rutgers University, Piscataway, NJ 08854, USA

## Abstract

Unlike other resident neural cells that are of neuroectodermal origin, microglia are resident neural cells of mesodermal origin. Traditionally recognized for their immune functions during disease, new roles are being attributed to these cells in the development and maintenance of the central nervous system (CNS) including specific communication with neurons. In this review, we highlight some of the recent findings on the bidirectional interaction between neurons and microglia. We discuss these interactions along two lines. First, we review data that suggest that microglial activity is modulated by neuronal signals, focusing on evidence that (i) neurons are capable of regulating microglial activation state and influence basal microglial activities; (ii) classic neurotransmitters affect microglial behavior; (iii) chemotactic signals attract microglia during acute neuronal injury. Next, we discuss some of the recent data on how microglia signal to neurons. Signaling mechanisms include (i) direct physical contact of microglial processes with neuronal elements; (ii) microglial regulation of neuronal synapse and circuit by fractalkine, complement, and DAP12 signaling. In addition, we discuss the use of microglial depletion strategies in studying the role of microglia in neuronal development and synaptic physiology. Deciphering the mechanisms of bidirectional microglial-neuronal communication provides novel insights in understanding microglial function in both the healthy and diseased brain.

## 1. Introduction

 Microglia comprise a unique subset of glial cells as the resident macrophages of the central nervous system (CNS). Although their developmental origin has been debated for several decades, the general contemporary consensus is that microglia originate from two sources to populate the CNS: an early source in the embryonic yolk sac and a later source from myeloid progenitors that invade the CNS during embryonic and postnatal development. Subsequent to this early colonization, the resident microglial population remains stable and is maintained through adulthood [1–3].

 Traditionally, microglia were studied for their role as pathologically responsive cells with virtually no interest in their functions in the healthy brain. However, given their presence in various species from the invertebrate leech to advanced mammals, as well as their emergence during early development, their functions cannot simply be restricted to pathological settings [4]. The last decade has witnessed a dramatic increase in microglia studies in the healthy brain. These studies suggest an interesting possibility that microglia and neurons engage in dynamic communication essential for nervous system development and maintenance. 

**Figure 1:**
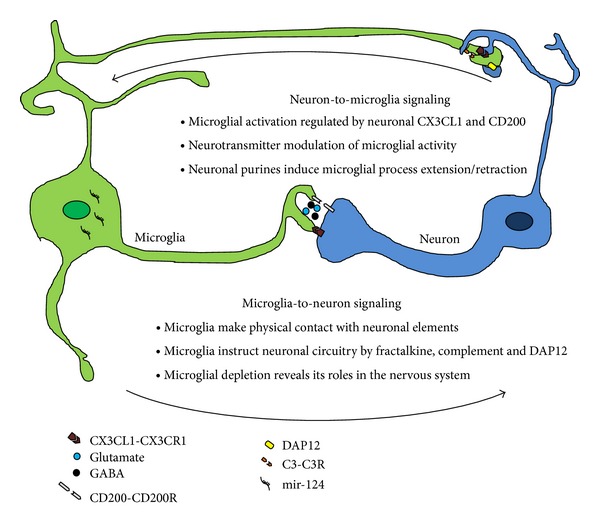
Bidirectional microglia-neuron communication in the healthy brain. Microglia-neuron interactions occur in both directions. Neurons can regulate microglial activation state through the unique ligand-receptor pairs (CX3CL1-CX3CR1 and CD200-CD200R), microRNA-124 (mir-124), neurotransmitters (glutamate and GABA), and purinergic signaling. Conversely, microglia also regulate neuronal activities. It is shown that the microglia is physically making contact with neuronal components. Moreover, fractalkine (CX3CL1-CX3CR1), complement (C3-CR3), and DAP12 signaling which occur distinctively between neurons and microglia are critical for the proper development and maintenance of neuronal circuits. Finally, the roles of microglia in the healthy brain are being elucidated by the several microglial depletion techniques.

 In the following pages, we survey the recent microglia literature that highlight the interaction between microglia and neurons in the healthy brain (Figure 1). Here, we emphasize the two-way communication that goes on between these cells, beginning with how neurons can modulate microglial state and activity, for example, by specific chemokine, classic neurotransmitters, and purinergic signaling. We then review the evidence that microglia, both by making direct physical contact with neuronal elements and releasing certain paracrine signals, can in turn alter neuronal behavior including the establishment of neuronal circuits. Moreover, we discuss microglial depletion studies as an approach to understand microglial importance in neuronal development, function, and maintenance (Figure 1).

## 2. The Neuron-to-Microglia Communication Axis

While the full repertoire of microglial functions in the CNS is yet to be elucidated, several reports have provided persuasive evidence that microglial functions are modulated by neuronal activities. These studies indicate that neurons can (a) regulate and/or maintain microglial activation states by secreting factors that influence basal microglial properties, (b) release neurotransmitters that influence microglial behavior, and (c) release purines that direct microglial chemotaxis during acute injury. Here, we perform a detailed review of neuronal-to-microglial signaling, though we recommend other reviews that have addressed the subject in detail in microglial-neuronal chemokine signaling [5, 6].

### 2.1. Neuronal Signals Regulate Microglial Activation State

One of the interesting microglial phenomena is that despite their exquisite sensitivity to perturbations in CNS homeostasis, by which they undergo rapid phenotypic and functional transformation into activated cells, they remain remarkably quiescent or “unactivated” while performing elaborate surveillance roles in the healthy brain. It is now recognized that this “unactivated” state is under the control, at least in part, of neuronal factors, including CD200 and fractalkine (CX3CL1). CD200 is a glycoprotein expressed on the neuronal cell surface in the CNS and functions by activating its receptor, CD200R, which is mainly expressed by microglia in the CNS parenchyma [7]. Genetic ablation of CD200 increased microglial activation, showing several molecular (increased expression of microglial activation markers such as CD11b and CD45) and morphological (decreased ramification) features of activation [8]. As with the above results, retinal microglia have also been shown to possess features of activation in CD200 knockout mice [9, 10]. These results suggest that neuronal CD200 acting through microglial CD200R keep microglia in a quiescent, unactivated state. Like the CD200-CD200R signaling axis, the CX3CL1-CX3CR1 signaling axis has also been implicated in the control of microglial activation state. CX3CL1 is expressed on neurons in the CNS, and CX3CR1 is expressed exclusively on microglia in the brain parenchyma. CX3CR1-deficient mice showed increased microglial cell-autonomous neurotoxicity in three different models of inflammation [11]. Therefore, neuronal release of CX3CL1 in the healthy brain may maintain microglia in a nonneurotoxic quiescent state. In addition, the CX3CR1 receptor seems to regulate microglial basal motility. For example, exogenous application of CX3CL1 resulted in increased motility in CX3CR1 heterozygotes but had no effect in knockouts suggesting that neuronal release of CX3CL1 not only maintains microglia in a quiescent state but also contributes to basal microglial surveillance. Consistently, microglial process dynamism is reduced in the CX3CR1 null but not CX3CR1 heterozygote mice in a retinal explant system [12]. The downstream effectors of microglial quiescence by neurons remain to be clarified. One intriguing possibility involves the regulation of microglia quiescence by microRNAs such as mir-124. Expression of this microRNA was found to distinguish CNS resident microglia from peripheral macrophages that expressed mir-223 but not mir-124 [13]. mir-124 was found to instruct microglial quiescence but was downregulated in activated microglia. Indeed, when neurons were cocultured with macrophages, mir-124 was upregulated and macrophages took on a more quiescent phenotype. Therefore, it is proposed that circulating blood cells with a macrophage phenotype migrate into the CNS during development and then gradually adopt a quiescent phenotype upon exposure to neuronal factors. Together, these studies suggest that as “transplants” into the CNS, microglia cells are “tamed” by signals from the principal elements of the nervous system.

Interestingly, in addition to keeping microglia in a quiescent state, neurons may also activate microglia during development likely with a different purpose. Neural precursor cells (NPCs) are present in certain neurogenic niches such as the hippocampal dentate gyrus. In such regions, microglia were recently shown to display more activated (as detected by CD68 expression) phenotypes [14]. The authors extended this correlation between NPC presence and microglial activation by *in vivo* NPC transplantation experiments. Here, NPCs were shown to increase microglial activation markers (e.g., CD68) in the striatum of injected mice. Moreover, the activated phenotype was reconstituted in mice injected with NPC conditioned media [14]. Specifically, NPC-derived vascular endothelial growth factor (VEGF) was found to be necessary and sufficient for the aforementioned phenotypic changes though the functional significance of the NPC-induced microglia activation is still largely unknown. 

### 2.2. Classic Neurotransmitters and Microglial Motility

A subject of increasing interest is the possibility that neuronal communication can also regulate microglial activity. However, whether microglia are capable of responding to neurotransmitters has only begun to be investigated in recent years, and the current data point to differences in global and local regulation of microglia activity by neurotransmitters. Before considering the primary evidence for modulation of microglial behaviors by specific neurotransmitters, we discuss three principal studies that showed that microglial activity is influenced by neuronal transmission. (i) Using two-photon imaging of neurons and microglia in the mouse visual cortex, Wake et al. [15] observed microglia making physical contact with neuronal elements. To test the requirement of neuronal activity for such contacts, the authors inhibited neuronal activity by either injecting TTX (which blocks sodium channels and thus action potentials) into the eye or lowering the body temperature of mice during imaging. Microglia displayed significantly reduced contacts with neurons under such conditions. (ii) Tremblay et al. [16] observed interaction between microglia and neuronal spines in the mouse visual cortex that was modified during visual experience. When mice were deprived of sensory input by dark adaptation, microglial motility was reduced and microglial processes were modified to display phagocytic structures suggesting the engulfment of material which may include neuronal elements. Interestingly, light reexposure restored microglial motility though phagocytic structures persisted. (iii) Most recently, neuronal activity-dependent microglial behaviors were reported in the zebrafish optic tectum [17]. As in the mouse [15, 16], the dynamic processes of zebrafish microglia made contacts with neurons through their bulbous endings. Additionally, when imaging was done in TTX, microglial bulbous endings were significantly reduced while repeated stimulation of a single eye by light resulted in increased microglial bulbous endings in the contralateral tectum [17]. Together these studies demonstrate that both physiological (e.g., eye deprivation or repetitive stimulation) and pharmacological (e.g., TTX application) alterations in neuronal activity can modulate microglial behavior. Regarding the modulation of microglial activity by neuronal activity, it should be pointed out that other studies reported that microglial sampling volume remained unaltered during TTX application *in vivo* [18] and microglial motility remained unchanged by neuronal activities induced by high frequency stimulation induced [19, 20].

Recent reports consistently suggest that global neurotransmission alters microglial motility. Using *in vivo* imaging in the mouse cortex, the first direct evidence showed that global inhibition of GABA-ergic neurotransmission resulted in an increase in the volume of tissue sampled by individual microglia [18]. Whether this effect is mediated by the direct action of inhibitory neurotransmitter on microglia or by other indirect factors like ATP released as a cotransmitter or secondary signal has yet to be clarified. Further evidence supported the role of global neurotransmission in regulating microglial activity in mouse retinal explants. As in the mouse cortex, retinal microglial morphological activity was increased by GABA inhibition. In addition, global inhibition of endogenous glutamatergic transmission decreased while exogenous glutamate receptor agonists increased microglial motility [21]. These authors suggested that neurotransmissional effects on microglial activity occurred through ATP. Together, these observations suggested that both excitatory and inhibitory neurotransmissions may act in concert to determine overall microglial activity. 

 Although the evidence for regulation of microglial motility by global levels of neuronal activity is mounting, the data for regulation of such activity by local neurotransmission is not as clear. Wu and Zhuo [20] first began to address this question by combining electrophysiology and imaging of resident microglia in brain slices. The authors found that local application of glutamate or GABA in the vicinity of ramified microglia did not induce membrane currents or chemotaxis of microglial processes. Moreover, activity-dependent synaptic plasticity induced by high-frequency stimulation failed to elicit changes in microglial motility. A subsequent study extended the observation in hippocampal slices to spinal cord slices as microglia failed to respond in a morphological significant way to a wide range of neurotransmitters by local application [19]. Despite these initial observations, recent *in vivo* imaging in the zebrafish optic tectum reported increased microglial activity to localized glutamate uncaging [17]. The differences may result from different methods of glutamate application, that is, through a pipette versus via uncaging, species differences, that is, mouse versus zebrafish, differences in tissue preparations that is, *in vivo* versus *ex vivo* slices, or differences in the age of tissue studied, that is, young animals (5–8 days post fertilization) versus adult (6–10 weeks old). This latter point is attractive since it has now been shown that even (astro) glial metabotropic glutamate signaling is developmentally regulated [22]. Moreover, like in the young zebrafish tectum, glutamate was found to elicit chemotactic responses from microglia in two-week-old spinal cord slices though the source of glutamate under such conditions remains to be determined [23]. 

In summary, although not conclusive, the current data suggest that microglial motility is decreased by global inhibitory neurotransmission and increased by excitatory neurotransmission, implying that such global communication between neurons also regulates the elaborate basal motile activity of microglia. Yet, whether neurotransmitter influence on microglial motility is developmentally regulated has not been clearly evidenced. In addition, although there has been interest in ionotropic receptors on tissue microglia (e.g., the reports by Fontainhas et al. [21] and Wu and Zhuo [20] failed to observe expression of functional glutamate receptors in microglia), further investigation is needed to determine the role of metabotropic glutamate/GABA receptors on tissue microglia and how they may integrate neuronal signals to modulate microglial functions. 

### 2.3. Traumatic Neuronal Signals “Activate” Microglia during Injury

**Figure 2:**
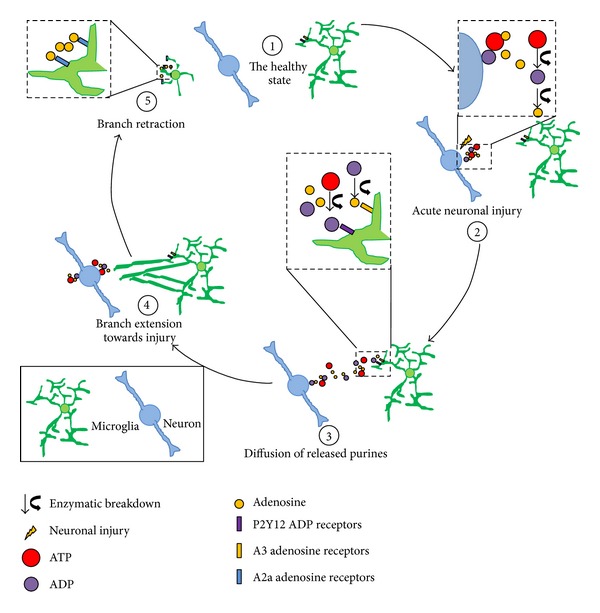
Neuron-to-microglia purinergic signaling regulates microglial extension and retraction. (1) In the healthy brain, microglia exist in close proximity to neurons. (2) In the event of neuronal injury, neurons release purines including ATP which can be degraded by endogenous enzymes into ADP and adenosine (magnification at top right). (3) Released purines diffuse in the extracellular space and can activate P1 (A3) and P2 (P2Y12) receptors on microglia that act in concert (magnification in center). (4) Purinergic activation leads to microglial branch extension towards the injury site. (5) Following microglial activation, adenosine can also activate A2a receptors that mediate microglial branch retraction.

 While clarity on neuron-to-microglia signaling via classic neurotransmitters during normal physiology is lacking, evidence for the communication during acute injury via purinergic signaling has been well established (Figure 2). We consider it in this review of microglia-neuron communication in the *healthy* brain because neuronal demise and the concomitant release of purines may occur physiologically, especially during development. However, the discussion here is also applicable during nervous system injury, disease, and pathology.

 Beginning with the work of Geoffrey Burnstock in the 1970s, a role for adenosine triphosphate ATP (which is a ubiquitous energy source) and its metabolites (e.g., adenosine diphosphate [ADP] and adenosine) in the extracellular space acting on cell surface receptors has now been established. These molecules are capable of eliciting different cell responses through two receptor types: ion channel P2X receptors that mediate ionic flux and G protein coupled P2Y receptors that activate G proteins and their downstream effectors (reviewed in [24]). Under normal physiological conditions, the concentrations of these metabolites are maintained at high levels within cells and at relatively lower levels in the extracellular space by a complex system that includes degrading enzymes, metabolite uptake, and generation of the respective purine [24]. However, during acute neuronal injury, these metabolites are released into the extracellular space at concentrations that could activate the respective receptor(s), many of which are expressed by microglia [25]. It is now clear that neuronal demise is detected and responded to by microglia in the event of the release of these purines. 

By the late 1990s, experiments with cultured microglia suggested that microglia expresses purinergic (P2) receptors as high concentrations of extracellular ATP induced intracellular Ca^2+^ elevation [26] and cell death [27] in a receptor-dependent manner. Further studies provided evidence for ATP- and ADP-induced microglial chemokinesis (motility) and chemotaxis (directed migration) in cultured microglia exposed to varying concentrations of purines [28]. Moreover, metabotropic purinergic (P2Y) receptors are implicated in the chemokinetic mechanism. Using *in vivo* imaging of microglial behavior, Davalos et al. [29] confirmed the relevance of ATP-induced microglial chemotaxis in the mouse cortex. The authors showed that laser-induced injury to brain tissue resulted in robust microglial branch extension towards the site of injury; the process chemotaxis was able to be abolished by apyrase, an ATP/ADP degrading enzyme. ATP-induced microglial chemotaxis was then confirmed in acute mouse brain slices and was further shown to involve ATP-induced outward potassium currents [30]. Using a similar acute brain slice preparation in rats, Kurpius et al. [31] also demonstrated that following tissue slicing, which inevitably induces neuronal injury, microglia cells are able to “home” rapidly to neuron-rich regions presumably by sensing endogenously released neuronal purinergic signals. Subsequently, ATP-induced microglial chemokinesis has been confirmed in the mouse spinal cord [19, 32] and retina [21] as well as in other animal models including the zebrafish [33] and leech [34] indicating the widespread existence of this signaling mechanism. The specific receptors involved in purine-induced chemotaxis have also been identified. Using a genetic approach, Haynes et al. [35] provided very powerful evidence *in vitro*, *ex vivo* (acute slice preparation), and *in vivo* for the regulation of ATP-induced microglial chemotaxis by the P2Y12 metabotropic receptor as branch extension or migration towards purinergic sources was abolished or significantly delayed in P2Y12 knockout microglia. Subsequently, using pharmacological approaches, Wu et al. [30] confirmed P2Y12 involvement in ATP-induced microglial chemotaxis in brain slice preparation.

As the forgoing has shown, ATP/ADP is capable of inducing microglial chemotaxis. However, whether ATP/ADP is sufficient for this cause has also been a topic of interest. It is well known that ATP and ADP are rapidly degraded by endogenous enzymes present in the extracellular space. The hypothesis that adenosine signaling may act in concert with P2Y12 signaling was first studied by Färber et al. [36]. The authors found that mice genetically deficient for a purine degrading enzyme that generates adenosine also showed deficient chemotaxis to ATP and ADP which was reconstituted by exogenous adenosine application. These results were extended *in vivo* in a focal ischemia model where microglia cells accumulate in neuron dense regions and microglial accumulation was significantly reduced in mice deficient in the ability to breakdown ATP/ADP to adenosine. More recently, the A3 adenosine receptor has been identified by Ohsawa et al. [37] using pharmacological approaches to be the specific adenosine receptor regulating microglial chemotaxis. A second adenosine receptor, the A2a receptor, has also been implicated in microglial process dynamics, and A2a receptor activation results in branch retraction [38]. 

Although purinergic signaling has been shown to be important for microglial motility during injury, an intriguing possibility exists that even in the healthy brain, physiological release of ATP is important. First, degradation of purines (ATP and ADP) by apyrase reduces basal microglial motility [31]. Moreover, zebrafish tectal neurons (but not microglia) were shown to express pannexin channels which release ATP upon glutamate uncaging suggesting that neurons may also communicate with microglia via purinergic signaling under physiological conditions [17]. Together, these results suggest that purinergic signaling is the most firmly established route of neuron-to-microglia signaling and may serve as a paramount mechanism by which the nervous system is maintained in a proper homeostatic state by microglia (Figure 2).

## 3. The Microglia-to-Neuron Communication Axis

By far, the literature is more extensive on the neuron-to-microglia communication axis in brain tissue or *in vivo*. Nevertheless interesting details are emerging on the specific ways in which microglia may instruct neuronal function. We consider this communication axis along three lines. First, we discuss some of the recent imaging evidence that microglia make direct physical contact with neurons which suggest neuro-modulatory roles for microglia during normal physiology. Next, we summarize some of the emerging data indicating that microglia regulate neuronal circuitry through identified signaling pathways, including fractalkine, complement receptor, and DAP12. Finally, we highlight some data that indirectly indicate the physiological role of microglia in neuronal function and synaptic activities by microglia depletion strategies.

### 3.1. Microglia Cells Make Transient Physiological Contact with Neurons

Microglia have been long recognized to make physical contact with phagocyte postmortem or dying neurons [39]. However, whether they make physical contact with healthy neurons would require the development of advanced imaging techniques. Live two-photon imaging in the uninjured mouse cortex already revealed that microglial processes were extremely dynamic and constantly undergoing remodeling by repeated branch extension and retraction [18, 29]. This remodeling was thought to be essential for microglial sensing of the microenvironment.

 Although microglial-to-neuronal soma contact in the living brain was observed by Nimmerjahn et al. [18], evidence for direct microglia-to-synaptic element contact in the living brain was first provided by Wake et al. [15] using two-photon imaging in the mouse cortex. The authors observed that, although seemingly undergoing random branch extension-retraction dynamics, microglial processes made direct and repeated contacts with dendritic spines. Interestingly, subsequent observations revealed that, in the developing brain, microglia-to-neuronal spine contacts were prolonged and microglia could modify the morphology of such spines during the third and fourth postnatal weeks [16, 40]. These results suggest direct microglia-to-synaptic element contact and a developmentally regulated mechanism in these interactions [15, 16]. Combining electron and two-photon microscopy, Tremblay et al. [16] were able to show that microglia indeed make contact with neurons, including synaptic spines, in the visual cortex in a manner that is dependent on visual experience. Moreover, similar interactions were reported in the both the visual and auditory cortices during adulthood and normal aging [41]. What then is the relevance of microglia-to-neuron contact? Wake et al. [15] found that following an hour of transient ischemia, microglia-to-neuron contact was prolonged from a duration of about 5 minutes in the healthy brain to about 80 minutes following transient ischemia and suggested that microglia function to monitor the functional state of neuronal synapses. In addition, work in the visual cortex showed that the physical contact of microglia with dendritic spines is able to alter the spine size in the healthy brain [16]. Microglial processes were found to preferentially localize to smaller dendritic spines that undergo the most dramatic changes in size during microglial contact. Moreover, chronic imaging indicated that a quarter of the microglia-contacted spines were eliminated over two days suggesting that microglia actively participate in the regulation of spine number and size in the healthy brain [16].

A more recent study in the zebrafish optic tectum also reported that microglial processes made bulbous contacts with neuronal soma under physiological conditions that increased with increasing neuronal activity [17]. Additionally, the authors reported that microglial processes made preferential contact with more active neurons as measured by calcium flux increases. Interestingly, microglia-contacted neurons displayed less activity following contact than non-contacted neurons, leading the authors to propose that a function of microglial-neuronal contact involves homeostatic mechanisms to downregulate neuronal excitability. However, future studies will have to identify the molecular mechanisms by which microglia “calm” excited neurons. 

### 3.2. Microglia Cells Contribute to Neuronal Circuitry Establishment

In addition to the possibility that microglia are involved in regulating acute neuronal activity by the physical contact of neurons, microglia cells are also important in the more long term wiring of neuronal circuits. Knockouts of specific microglial receptors have now been studied to determine the contribution of microglia to neuronal circuitry development. Here, the emerging picture indicates that microglia cells are cellular components that participate in establishing functional neuronal circuits through several molecular pathways, including fractalkine receptor, complement receptor, and DAP12.

A recent study showed that fractalkine receptor knockouts displayed a transient reduction in hippocampal microglial numbers from the beginning of the second through to the end of the fourth postnatal week [42]. In the same mice, the authors observed increased dendritic spine density on hippocampal neurons during the second postnatal week [42]. High resolution microscopy data further showed microglia phagocyte synaptic components and that the increased spine density in fractalkine knockout mice is likely due to a defect in microglial phagocytosis of synapses during development. Evidence for microglial engagement with synaptic components using high-resolution microscopy has also been documented by other studies [16, 41, 43]. These initial reports on defective neuronal development in the hippocampus of fractalkine receptor-deficient mice were extended to the cortex in another study where proper maturation of thalamocortical circuits in the barrel cortex of developing mice was shown to require functional fractalkine signaling [44]. The behavioral consequence of microglial fractalkine signaling has also been investigated. Rogers et al. [45] showed that even the loss of a single functional fractalkine receptor allele resulted in significant deficiencies in motor learning, contextual fear, and memory. These behavioral observations were correlated with an impairment in cellular LTP, modulated molecularly by an increase in proinflammatory IL-1*β* release, and activity in fractalkine receptor heterozygotes and homozygotes relative to wildtypes. Moreover, this signaling axis has been shown to be important in mediating the enhancement of synaptic plasticity and spatial memory induced by rearing in an enriched environment [46]. 

As with fractalkine signaling, complement signaling has been reported in microglial pruning of developing neuronal synapses. During development, extranumerary synapses are pruned in the functional development of the nervous system. Microglia have now been identified in the process of synapse elimination, a process that involves neuronal communication to microglia via complement signaling. First, it was identified that C1q, an upstream member of the complement signaling cascade, colocalized with synapses in the developing CNS. Interestingly, genetic ablation of C1q resulted in an excess number of synapses during adolescence, a result that was recapitulated in mice deficient with C3, a downstream member of the complement cascade [47]. To determine the mechanism of elimination, a follow-up study further showed that microglia engulfed synaptic material in a C3-receptor-dependent manner during early postnatal development [43]. Thus, synapses to be eliminated were proposed to be tagged for elimination by complement proteins which serve as a signal to microglia for engulfment and subsequent elimination. 

Apart from the fractalkine and complement signaling axes, a role for microglial DAP12 has also been reported in the development of functional neuronal synapses. DAP12, expressed mainly on hematopoietic cells, was shown to be exclusively expressed on microglia in the hippocampus around birth [48]. Intriguingly, in genetically deficient DAP12 mice, developmental apoptosis of neurons was decreased [49] but synaptic plasticity was enhanced [48]. Interestingly, DAP12 function has also been linked to TREM2, a known regulator of microglial phagocytosis [50, 51]. Another mechanism of DAP12's regulation of synaptic plasticity was suggested to involve brain derived neurotrophic factor (BDNF) signaling via its receptor (TrkB) on neurons as DAP12 deficient mice also had reduced TrkB expression at synaptic sites. DAP12 contains a tyrosine-based motif, a docking site for Syk tyrosine kinases, promoting activation of PI3 K and ERK pathways [52]. How these signaling pathways are coupled to BDNF pathway needs to be further investigated. 

Microglial roles in normal neuronal circuitry are becoming increasingly appreciated from several recent studies correlating microglial function with behavior. For example, aberrant microglia cells were shown to be responsible for pathological grooming behavior in mice and could be rescued by bone marrow transplantation [53]. Similarly, microglial dysfunction, especially in phagocytosis, was implicated in a mouse model for Rett syndrome, an autism spectrum disorder [54]. Within the context of normal development, microglia cells were instructive in the determination of masculine features and behavior in developing rats. Here, compared to females, microglial numbers were significantly increased in the male preoptic area which is responsible for sex-specific development. Interestingly, the male preoptic area also had an increase in dendritic spines suggesting that microglia may actually stabilize existing and/or induce the formation of dendritic spines [55]. 

### 3.3. Microglial Depletion and Its Neuronal Effects

A useful approach to gain insights into microglial modulation of neuronal activity is to perform microglia depletion experiments. Currently, there are five methods being used for the ablation of microglia *in vivo* or in cultured brain slices. (1) One method involves the use of CD11b-HSVTK mice in which the herpes simplex virus thymidine kinase (HSVTK) is placed under the control of the CD11b promoter expressed exclusively by microglia in the brain [56]. Thymidine kinase converts ganciclovir into cytotoxic kinases leading to cell suicide. Thus, ganciclovir exposure can serve as an inducible cell suicide technique in HSVTK-expressing microglial cells in transgenic CD11b-HSVTK mice. (2) A second method involves the use of DTR mice in which the human dipthera toxin receptor (DTR) is expressed under the control of CD11b [57]. Human DTR expressing microglial cells can be selectively ablated by localized injection of the dipthera toxin in the mice. (3) A third method involves the use of PU.1 knockout mice that lack the PU.1 hematopoietic-lineage specific transcription factor resulting in a lack of mature hematopoietic cells including microglia [58, 59]. However, the mice die by late gestation or shortly after birth. (4) A fourth method involves the use of CSF1R knockout mice. CSF1R knockout mice in which a null mutation in a macrophage-specific receptor, the colony stimulating factor-1 receptor (CSF1R), results in effective elimination of brain microglia embryonically and postnatally [60]. Interestingly, these mice are viable for up to the third postnatal week. (5) The last method involves ablation using clodronate. Clondronate can function as an intracellular mediator of apoptosis. Once encapsulated in liposomes, they can be engulfed by phagocytes that degrade the liposomes to release its contents. This approach was first used in selectively depleting macrophages [61] but has now been applied to microglia ablation [62–64]. Clondronate has so far been used extensively in slice cultures and has only recently been employed for microglial depletion in the embryonic [62] and neonatal [63] brain. However, whether this method can be used in the adult brain to elucidate microglial roles in the healthy brain remains to be determined. 

Although microglial ablation studies have been directed towards effects on disease progression, including multiple sclerosis [56], ischemic stroke [65], Alzheimer's disease [66, 67], ALS [68], epilepsy [69], bacterial meningitis [70], and brain tumor [71], the discussion of microglia in brain disease is beyond the scope of the current review. Therefore, we will mainly focus on recent studies using the microglia ablation strategy to gain important insights on the role of microglia in normal physiology, including brain development and synaptic transmission. 

Microglia depletion studies showed that microglia is critical for brain development. By using the clondronate liposome ablation method, microglia can be effectively eliminated from neonatal cerebellar slices within three days [72]. Microglial depletion in this fashion prevented the death of Purkinje neurons suggesting that microglia at this early postnatal period are active in developmental neuronal death. The mechanism involves the activation of NADPH oxidase and the release of reactive oxygen species in killing neurons. The notion of microglia in brain development was further supported by a recent study using CSF1R knockout mice [60]. These mice were devoid of microglia by 65–99% depending on brain region. In the postnatal knockout mice, there are defective brain structures including enlarged ventricles and compressed parenchyma in the olfactory bulb and cortex. Therefore, the results indicate the critical role of microglia in the maintenance of brain architecture during development; though since CSFR is also expressed in the periphery, a role for cells outside the CNS has not been ruled out. 

Using microglia ablation strategies, recent studies also shed new light on the role of microglia in synaptic transmission. Pascual et al. [73] investigated the effects of microglial depletion on synaptic transmission using PU.1 knockout mice. Given the late embryonic or early postnatal lethality of mice with a disrupted PU.1 gene, the authors cultured hippocampal slices from PU.1 knockout mice at birth. As expected, microglia were lacking in these mice even after 10–14 days in culture. Interestingly, spontaneous neuronal activity in the form of excitatory postsynaptic potentials (EPSPs) remained unchanged between microglial “wildtype” and “knockout” slices. However, in the presence of inflammatory stimuli, such as LPS, microglia were able to alter neuronal EPSPs within minutes which was absent in slices lacking microglia. The mechanism involves ATP release from microglia and the participation of astrocyte in the modulation of synaptic transmission. These results indicate that microglia are able to directly sense inflammatory signals and rapidly translate that information indirectly to neuronal physiology. Another recent study has incorporated the clondronate ablation approach to provide insights to microglial regulation of neuronal activity. Using an organotypic hippocampal slice culture preparation with clondronate ablation of microglia, Ji et al. [64] reported that, in the absence of microglia, hippocampal neurons exhibited enhanced frequency of synaptic currents, while replenishment of microglia reverses the effect of microglial depletion on synaptic functions, suggesting that microglia reduce or properly “tune” synaptic activity. Consistently, culturing neurons in the presence of microglia resulted in a reduction in the number of synapses [64]. The study corroborates results from earlier studies that demonstrated microglial engulfment of synaptic material during early postnatal development of the murine hippocampus and lateral geniculate nucleus [42, 43]. It is worth noting that the studies by Pascual et al. [73] and Ji et al. [64] reported conflicting consequences to the absence of microglia on basal synaptic activity. Although it is not entirely clear why this is the case, it is possible that the discrepancy may be due to the different methods of microglial depletion and the timing of depletion. In summary, several approaches (pharmacological and genetic) are now available to begin to address neurotransmission in the absence of microglia. Thus far, the modulatory role of microglia in neurotransmission remains a poorly investigated endeavor but is sure to uncover novel insights into microglial functions in the nervous system. However, it should be kept in mind that depletion strategies that result in microglial death may alter the neural tissue milieus inadvertently altering neuronal activity. Therefore, data achieved using microglia depletion studies should be received with caution and performed perhaps with complementary approaches. 

## 4. Conclusion

 The role of microglia during injury and disease has been studied for a few decades. However, the dynamics of microglia-neuron communication in the healthy brain have only gained attention in recent years with already interesting results. The current evidence indicates that the communication between microglia and neurons is bidirectional involving several immunomodulatory factors and signaling axes including purinergic, neurotransmitter, chemokine, and complement signaling. Yet, many unanswered questions remain including the repertoire of microglial functions in the healthy brain during neural development and maintenance, the role of neurotransmitter signaling on microglial activity, and the immediate consequence of microglial engagement with synaptic elements making this field of research a veritable treasure throve in the next decade.
